# Prevalencia de anomalías dentarias en radiografías panorámicas de pacientes entre 10 y 30 años de un centro radiográfico. un estudio transversal

**DOI:** 10.21142/2523-2754-1301-2025-231

**Published:** 2025-03-03

**Authors:** Sergio Ricardo Escuza Gonzalez, Axel Carlos Díaz Álvarez, Carlos Vigo García

**Affiliations:** 1 Carrera de Estomatología, Universidad Científica del Sur. Lima, Perú. sergioricardoeg@gmail.com diazalvarezaxel@gmail.com cvigo@cientifica.edu.pe Universidad Científica del Sur Carrera de Estomatología Universidad Científica del Sur Lima Peru sergioricardoeg@gmail.com diazalvarezaxel@gmail.com cvigo@cientifica.edu.pe

**Keywords:** radiografía panorámica, anomalías dentarias, epidemiología, panoramic radiograph, dental anomalies, epidemiology

## Abstract

**Introducción:**

Las anomalías dentarias están asociadas con problemas en el desarrollo, lo que afecta estructuras faciales y dentales con potenciales repercusiones funcionales, oclusales y estéticas. La detección temprana mediante radiografías panorámicas es crucial para un tratamiento y manejo adecuado de estas alteraciones.

**Objetivo:**

Determinar la prevalencia de anomalías dentarias en radiografías en pacientes atendidos en un centro radiológico durante el 2023.

**Materiales y métodos:**

Se realizó un estudio retrospectivo, observacional y descriptivo, en el que se analizó 1500 radiografías panorámicas. Se empleó un método de muestreo aleatorio simple y se evaluaron diversas variables, como tipos y número de anomalías, edad, género y ubicación en los cuadrantes bucales. Se utilizó el programa Romexis Viewer para el análisis de imágenes.

**Resultados:**

En la prevalencia de anomalías dentarias de estructura, se observó que la hipoplasia del esmalte y la hipercementosis presentaron una menor cantidad de casos, con una prevalencia del 1%. En las anomalías de tamaño, se encontró que la microdoncia tuvo una prevalencia del 10,5%. También, en las anomalías de erupción, se observó que la posición ectópica afectó al 6,7% de los casos. Por otro lado, en las anomalías de número, se observó que la agenesia tuvo una prevalencia del 5,2%. En las anomalías de morfología no hubo casos de unión, mientras que la dilaceración estuvo presente en el 58,6% de los casos. La rizomicri tuvo una prevalencia del 12,4%.

**Conclusiones:**

Las anomalías dentarias en las radiografías de los pacientes evaluados tienen prevalencias variables. El estudio reafirma la importancia de la radiografía panorámica en la detección temprana de anomalías dentarias, crucial para la planificación de un tratamiento adecuado y personalizado en odontología.

## ASBTRACT

**Introduction:**

Dental anomalies are associated with developmental problems, affecting facial and dental structures with potential functional, occlusal and aesthetic repercussions. Early detection using panoramic radiographs is crucial for adequate treatment and management of these alterations. Objective: Determine the prevalence of dental anomalies in radiographs in patients treated in a radiological center during the year 2023.

**Materials and Methods:**

a retrospective, observational and descriptive study was carried out, analyzing 1500 panoramic radiographs. A simple random sampling method was used and various variables such as types and number of anomalies, age, gender, and location in the oral quadrants were evaluated. The Romexis Viewer program was used for image analysis.

**Results:**

In the prevalence of dental anomalies of structure, it was observed that enamel hypoplasia and hypercementosis presented a lower number of cases, with a prevalence of 1%. In size anomalies, it was found that microdontia had a prevalence of 10.5%. Also, in eruption anomalies, it was observed that the ectopic position affected 6.7% of cases. On the other hand, in number anomalies, it was observed that agenesis had a prevalence of 5.2%. In morphology anomalies, there were no cases of union, while dilaceration was present in 58.6% of cases. Rhizomicri had a prevalence of 12.4%.

**Conclusions:**

Dental anomalies observed in the radiographs of the evaluated patients show variable prevalence rates. The study reaffirms the importance of panoramic radiography in the early detection of dental anomalies, crucial for planning adequate and personalized treatment in dentistry.

## INTRODUCCIÓN

Las anomalías dentarias, reflejo de problemas en el desarrollo a lo largo del tiempo, pueden afectar directamente las estructuras faciales y la dentición, con el potencial de generar futuras patologías ^1^. Estas alteraciones suelen perturbar las relaciones morfológicas naturales del cráneo y la mandíbula, con repercusiones en aspectos funcionales, oclusales y estéticos ^2^^,^^3^. La detección precoz permite intervenciones tempranas y la prevención de trastornos más graves. Por tanto, las radiografías panorámicas, esenciales en el diagnóstico odontológico, evalúan los tejidos duros y, ocasionalmente, detectan patologías en tejidos blandos ^4^^,^^5^, y así orientan al odontólogo en el tratamiento adecuado ^(6, 7)^. Estas alteraciones evidenciadas se manifiestan como deformaciones o cambios estructurales ^8^^-^^10^, con raíces genéticas, congénitas y ambientales, que pueden causar trastornos en el desarrollo dentario y afectar la morfología, tamaño y cantidad de piezas dentales, así como el desarrollo de tejidos adyacentes ^11^^-^^14^. A menudo, se presentan en niños, generando problemas como dolor, infección y dificultades funcionales ^15^^-^^18^. Comprender su prevalencia es esencial para identificar causas, tratamientos y estrategias preventivas eficaces ^19^.

La práctica y análisis de interpretación de radiografías panorámicas es fundamental para el profesional en estomatología, dado que el margen de error está estrechamente ligado a los conocimientos del operador ^20^^,^^21^. Se estima que el promedio de error oscila entre el 19% y el 41% ^18^. Por ello, esta investigación fue realizada con el objetivo de recabar la mayoría de los hallazgos presentes en las radiografías panorámicas, analizar la información obtenida y lograr una conclusión acerca de la prevalencia de anomalías dentales, debido a la necesidad de una intervención temprana y la prevención. Al comprender estos problemas, los profesionales de atención odontológica pueden garantizar una salud bucal óptima para los pacientes y mejorar su calidad de vida en general.

## MATERIALES Y MÉTODOS

Para la ejecución de esta investigación, se obtuvo la aprobación del Comité de ética e investigación de la Universidad Científica del Sur, el cual obtuvo el código de registro N.° PRE-8-2023-00444. Este estudio fue retrospectivo, observacional y transversal, en el cual se identificaron anomalías dentarias mediante el estudio de radiografías registradas anteriormente en las instalaciones de un centro radiológico durante e 2023, de pacientes entre los 10 y 30 años. La población estuvo conformada por 1500 radiografías panorámicas similares al estudio de Bilge NH ^22^, obtenidas mediante un equipo de rayos X dental panorámico y cefalométrico.

Se escogió este intervalo de edades ya que se pueden encontrar anomalías desde la erupción de las piezas en pacientes jóvenes justo durante el periodo de erupción hasta los 30 años, que es aproximadamente la edad máxima en la que se frecuentan las anomalías dentarias ^22^. La muestra se estimó empleando una fórmula para aproximar la medida requerida para el estudio, que será aproximadamente de 180; pero al tomar en cuenta el porcentaje esperado de pérdidas, el tamaño de la muestra final fue de 210. Para la estimación de este tamaño de muestra se empleó una proporción del 5% de significancia, ya que tener un nivel de confianza del 95% y un 5% de significancia es lo estándar, pues proporciona un buen balance entre la precisión y la amplitud del intervalo de confianza ^23^. El tipo de muestreo empleado fue de tipo aleatorio simple y se empleó una lista de base de datos confeccionada sobre un Excel acerca de las radiografías panorámicas del centro radiográfico con los datos en el rango de la edad objetiva. 

Entre los criterios de inclusión se incluyeron radiografías panorámicas de pacientes de ambos géneros y radiografías panorámicas en óptimas condiciones para su estudio. Se excluyeron las radiografías panorámicas con hallazgos que dificulten realizar un correcto análisis y radiografías panorámicas con presencia de objetos o interferencias en la imagen. Sobre las variables y operacionalización, la variable principal fue la presencia de anomalías dentarias. La existencia de alguno de los tipos de anomalías dentarias en la radiografía panorámica se obtuvo mediante la observación directa. Las covariables fueron los tipos de anomalías dentarias, los cuales se hallaron mediante la observación directa en la investigación, comenzando por el número de anomalías dentarias, la cuales se cuantifican según los hallazgos por radiografía del paciente; la edad, el cual fue tomado en cuenta desde el nacimiento hasta el momento de referencia; el género, el cual se rigió según las características biológicas y anatómicas, y fue descrito por la leyenda de la radiografía, cuyas opciones son masculino o femenino. Por último, la posición, la cual se agrupó según la ubicación del defecto dentro de los cuadrantes de la cavidad oral. Esta se obtuvo mediante la observación directa y podrá ser uno de los cuatro cuadrantes. Se utilizó como método la observación estructurada. Se empleó el programa Romexis Viewer v6.4.4.7.12, utilizando las herramientas de procesamiento de imágenes y los filtros proporcionados por el *software*. El procedimiento inició solicitando la autorización y acceso mediante una carta simple al centro radiológico para la obtención y el procesamiento de las radiografías panorámicas. La obtención de radiografías en cuestión fue tomada con un equipo NewTom GIANO HR, un equipo radiográfico multifuncional para una gama completa de exámenes 2D y 3D dentales. Este posee un voltaje de ánodo fijo entre 60 kV y 85 kV. Una corriente de ánodo entre 2 mA y 16 mA; un tiempo de exposición de hasta 12,3 s en adultos y 11,2 s en niños; un factor de ampliación de 1,25; una resolución máxima de 6,3-7,5 lp/mm y un tamaño de píxel de 70-80 um; un tamaño máximo de 27 x 15 cm para adultos y 22 x 13 para niños. 

Para la valoración de imágenes correspondientes a las radiografías panorámicas fueron exportadas como archivos de imagen y evaluadas en un monitor con iluminación moderada constante mediante observación directa en el programa de Romexis Viewer. En primer lugar, se evaluó la calidad de la radiografía considerando la toma, el contraste y la exposición, verificando que estén dentro de los márgenes permisibles para su evaluación. Posteriormente, se procedió con la evaluación lógica de la radiografía utilizando el método de la espiral decreciente. Se comenzó por el cóndilo derecho y se observó hasta llegar al borde inferior de la mandíbula; luego, se continuó hacia el cóndilo izquierdo. Posteriormente, se examinó el maxilar, verificando los senos maxilares y el paladar duro, y se continuó por la escotadura sigmoidea derecha, rodeando la mandíbula y observando el periodonto y el hueso alveolar. Desde la escotadura sigmoidea izquierda, se siguió el mismo procedimiento, pero observando el periodonto y el hueso alveolar del maxilar. Finalmente, se analizaron cada uno de los dientes y el hueso alveolar. Para concluir, se comparó cualquier alteración encontrada con la anatomía normal ^24^.

Para asegurar la calidad del estudio, se llevó a cabo una prueba piloto con el 10% de la muestra propuesta según el estudio de Bilge NH ^22^. Este proceso se realizó con el fin de garantizar una adecuada capacitación y calibración de los investigadores. La prueba piloto se ejecutó utilizando el mismo formato que en el proyecto final y fue supervisada por un radiólogo experto con varios años de experiencia (CVG). Se empleó una base de datos proporcionada por CDI Perú y se llevó a cabo mediante la observación de radiografías panorámicas y la verificación de los datos correspondientes. Este proceso se repitió hasta obtener un índice de coeficiente de correlación intraclase superior a 0,80 tanto para la evaluación entre observadores como para la evaluación de un mismo observador en diferentes momentos. Todos los resultados obtenidos se registraron en una hoja de recolección de datos en formato Excel.

### Análisis estadístico

Se confeccionó una base de datos en Excel de los resultados evidenciados sobre la prevalencia de anomalías dentarias del CDI. Los resultados fueron examinados mediante estadística descriptiva mediante el empleo del *software* SPSS versión 26 (IMB, EE. UU.). Se emplearon las medidas descriptivas centralizadas con entrecruzamiento y medidas descriptivas de dispersión. Se empleó la varianza, desviación típica y desviación media. Finalmente, se realizó una prueba estadística de chi-cuadrado de Pearson. Se trabajo con un p < 0,05.

## RESULTADOS

La prevalencia de anomalías dentarias según su morfología mostró que la dilaceración fue bastante común (58,6%). Los *dens in dente* estuvieron en 1,9% y el diente evaginado apareció en el 30%. Las raíces supernumerarias se encontraron en el 21.9% de los casos, y el diente en pala en el 15.2%. La prevalencia de anomalías dentarias según su tamaño mostró la microdoncia con un 10,5%; la macrodoncia, con un 1,0%; rizomicri, con un 12,4%, y rizomegalia, con un 2,9% (Tabla 1). Asimismo, se evaluó la prevalencia de anomalías dentarias según su erupción estando la posición ectópica en un 6,7%, los diastemas estuvieron en un 4,3%, y las giroversiones, en un 60,5% (Tabla 2). Por otro lado, la prevalencia de anomalías dentarias según su número mostró agenesia en un 5,2% e hiperodoncia en un 4,8%. Las anomalías dentarias según su estructura muestran a la hipoplasia del esmalte y la hipercementosis como las únicas anomalías presentes en un pequeño porcentaje de casos, cada una con una prevalencia del 1% (Tabla 2).

**Tabla 1:** Prevalencia de anomalías dentarias en radiografías según forma y tamaño

	No presenta		Presenta		Total	
	n	%	n	%	n	%
Alteración de forma						
Unión	210	100,00%	0	0,00%	210	100%
Dilaceración	87	41,40%	123	58,60%	210	100%
Dens in dente	206	98,10%	4	1,90%	210	100%
Diente evaginado	147	70,00%	63	30,00%	210	100%
Perlas del esmalte	208	99,00%	2	1,00%	210	100%
Taurodontismo	193	91,90%	17	8,10%	210	100%
Raíz supernumeraria	164	78,10%	46	21,90%	210	100%
Diente en pala	178	84,80%	32	15,20%	210	100%
Alteración de tamaño						
Microdoncia	188	89,50%	22	10,50%	210	100%
Macrodoncia	208	99,00%	2	1,00%	210	100%
Rizomicri	184	87,60%	26	12,40%	210	100%
Rizomegalia	204	97,10%	6	2,90%	210	100%

**Tabla 2:** Prevalencia de anomalías dentarias en radiografías según erupción, número y estructura

	No presenta		Presenta		Total	
	n	%	n	%	n	%
Alteración de erupción						
Posición ectópica	196	93,30%	14	6,70%	210	100%
Diastemas	201	95,70%	9	4,30%	210	100%
Giroversiones	83	39,50%	127	60,50%	210	100%
Inclinaciones	107	51,00%	103	49,00%	210	100%
Diente sumergido	209	99,50%	1	0,50%	210	100%
Inclusión	144	68,60%	66	31,40%	210	100%
Impactación	132	62,90%	78	37,10%	210	100%
Retención	175	83,30%	35	16,70%	210	100%
Alteración de número						
Agenesia	199	94,80%	11	5,20%	210	100%
Hiperodoncia	200	95,20%	10	4,80%	210	100%
Alteración de estructura						
Hipoplasia del esmalte	208	99%	2	1%	210	100%
Amelogénesis imperfecta	210	100%	0	0%	210	100%
Dentinogénesis imperfecta	210	100%	0	0%	210	100%
Displasia dentinal	210	100%	0	0%	210	100%
Hipercementosis	208	99%	2	1%	210	100%
Odontodisplasia regional	210	100%	0	0%	210	100%

Con respecto al número de anomalías dentarias por paciente, se observó que las alteraciones de forma fueron las más frecuentes, con una media de 3,36 ± 2,32. Las alteraciones de erupción tuvieron una media de 4,18 ± 2,71, y las alteraciones de estructura fueron las menos comunes, con una media de 0,02 ± 0,14 (Tabla 3). Las anomalías dentarias estuvieron distribuidas entre los diferentes cuadrantes, con una mayor prevalencia observada en el cuarto cuadrante (Tabla 4).

**Tabla 3:** Número de anomalías dentarias en las radiografías panorámicas

Número de alteraciones dentarias	Media	Desviación estándar	Mínimo	Máximo
Forma	3,36	2,32	0	11
Tamaño	0,64	1,62	0	11
Erupción	4,18	2,71	0	13
Número	0,25	1,59	0	22
Estructura	0,02	0,14	0	1
Total	8,45	4,17	1	30

**Tabla 4:** Frecuencia de anomalías dentarias según el cuadrante en el que se ubican

Cuadrante	No presenta		Presenta		Total	
	n	%	n	%	n	%
Primer cuadrante	27	12,9%	183	87,1%	210	100%
Segundo cuadrante	24	11,4%	186	88,6%	210	100%
Tercer cuadrante	23	11,0%	187	89,0%	210	100%
Cuarto cuadrante	18	8,6%	192	91,4%	210	100%

El grupo de 10 a 15 años tuvo la mayor frecuencia, con el 30,5% del total de anomalías. Le siguió el grupo de 16 a 20 años, con un 25,2%, y luego el grupo de 21 a 25 años, con un 24,3% (Tabla 5). Acerca de las anomalías dentarias según el género de los pacientes, se observó que todos los pacientes presentaron alguna anomalía dentaria, ya que no se registraron casos sin anomalías en ninguno de los géneros. El 43,3% de las anomalías se encontró en pacientes masculinos, mientras que el 56,7% se presentó en pacientes femeninos. No se pudo determinar el valor p de la prueba chi-cuadrado por estar la prevalencia de anomalías presente en toda la muestra (Tabla 6).

**Tabla 5:** Frecuencia de anomalías dentarias según la edad de los pacientes.

Edad	Anomalías						P-valor
	No presenta		Presenta		Total			
	n	%	n	%	n	%	
De 10 a 15 años	0	0,0%	64	30,5%	64	30,5%	N. D.
De 16 a 20 años	0	0,0%	53	25,2%	53	25,2%	
De 21 a 25 años	0	0,0%	51	24,3%	51	24,3%	
De 26 a 30 años	0	0,0%	42	20,0%	42	20,0%	
Total	0	0,0%	210	100,0%	210	100,0%	

N. D.: no determinado, chi cuadrado

**Tabla 6:** Frecuencia de anomalías dentarias según el género de los pacientes

Sexo	Anomalías						P-valor
	No presenta		Presenta		Total			
	f	%	f	%	f	%	
Masculino	0	0,0%	91	43,3%	91	43,3%	N. D.
Femenino	0	0,0%	119	56,7%	119	56,7%	
Total	0	0,0%	210	100%	210	100%	

N. D.: no determinado, chi cuadrado

## DISCUSIÓN

Existe presencia de anomalías dentarias en la totalidad de las radiografías evaluadas para un diagnóstico dental. Cada tipo de anomalía requiere un enfoque de manejo único debido a su potencial de impacto en la estética, función y salud bucodental. Entre ellas se destacan principalmente las relacionadas con la erupción y la forma de los dientes. Las variaciones individuales de cada diente van a aumentar la probabilidad de encontrar dientes mal posicionados o con características anatómicas distintas. En el presente estudio, el grupo de 10 a 15 años tuvo la mayor frecuencia (30,5%), seguido por los grupos de 16 a 20 años (25,2%), 21 a 25 años (24,3%), y 26 a 30 años (20,0%). La prevalencia fue mayor en los pacientes más jóvenes. El 43,3% de las anomalías se observó en hombres y el 56,7%, en mujeres, siendo más prevalentes en el género femenino. Aboujaoude *et al*. ^25^ corroboran, con su estudio realizado en pacientes pediátricos libaneses, que existe mayor prevalencia de anomalías dentarias en pacientes entre 10,5 ± 1,8. Además, Naccha *et al*. ^26^ indican también una mayor prevalencia de anomalías dentarias en el grupo de pacientes entre los 13 y 15 años, con un 52,9%, además de una mayor incidencia en pacientes femeninas. 

Da Silva *et al*. ^27^, en su estudio realizado en pacientes entre 6 y 50 años en Brasil, indican que factores como la edad del paciente, la predisposición genética, el ambiente, el desarrollo y las condiciones de salud influyen en la incidencia y gravedad de estas condiciones. Además, indican que existe prevalencia en anomalías de número con las agenesias en pacientes entre 11 y 15 años, con una prevalencia del 27,86%, lo que contrasta con los hallazgos de este estudio, donde se evidenciaron agenesias solo en el 5,2% de los casos. 

León *et al*. ^28^, en su estudio realizado en pacientes de 6 a 17 años en la ciudad de Huánuco (Perú), evidencian una prevalencia significativa de malformaciones en forma y tamaño dental en infantes con un 85,1% y un 14,9%, respectivamente, y que la anomalía de forma es la más frecuente. Esto le brinda soporte al presente estudio, ya que una de las anomalías más frecuentes evidenciadas fue la de forma, comenzando por las dilaceraciones (58,6%), seguidas por los dientes evaginados (30%) y las raíces supernumerarias (21,9%). Este estudio también evidenció la prevalencia en anomalías de erupción, con las giroversiones como las más prevalentes (60,5%), seguidas por las inclinaciones (49%), la impactación (37,1%), la inclusión (31,4%), las retenciones (16,7%) y las posiciones ectópicas (6,7%). Esto concuerda con el estudio realizado por Vinjolli *et al*. ^29^ en pacientes albaneses de entre 8 y 30 años, entre los cuales hallaron mayor prevalencia en anomalías de erupción como impactación dental (7,6%) y posición ectópica (5,3%). Amado *et al*. ^30^, en su estudio en pacientes argentinos de 6 a 15 años, también registró una mayor prevalencia de anomalías de erupción sobre piezas dentarias retenidas en un 44,22% de los casos; esto contrasta con lo obtenido en el presente estudio, la cual fue de mucha menor incidencia. 

Por último, entre las limitaciones evidenciadas durante esta investigación resalta que los resultados no se pueden generalizar y, principalmente, tienen validez interna. Finalmente, esta investigación reafirma la importancia de la radiografía panorámica en la detección temprana de anomalías dentarias, lo que subraya su papel crucial para la planificación de un tratamiento adecuado y personalizado en odontología. Al ofrecer una visión detallada de la estructura dental y los tejidos circundantes, permite a los profesionales identificar problemas potenciales en una etapa inicial. Esto facilita la implementación de estrategias preventivas y el diseño de planes de tratamiento precisos y adaptados a cada paciente, lo cual mejora los resultados de salud dental a largo plazo.

## CONCLUSIONES

En este estudio se demostró una mayor prevalencia de anomalías dentarias en pacientes de 10 a 15 años, con un predominio del sexo femenino. Este estudio refuerza la importancia de una formación especializada y un conocimiento técnico sólido en la interpretación de radiografías, lo que resalta la relación entre la precisión del diagnóstico y el conocimiento del operador. La identificación temprana de estas anomalías dentarias es fundamental para la implementación de intervenciones preventivas y personalizadas que contribuyan no solo a una salud bucal óptima de los pacientes, sino también a una mejor calidad de vida. Esto subraya la necesidad del profesional de prestar más atención durante su práctica clínica a dichas anomalías al realizar un análisis radiográfico y así mejorar los resultados y reducir complicaciones futuras en los pacientes.

## Financiamiento:

El siguiente proyecto no cuenta con ningún apoyo financiero
